# Incidence of lower respiratory tract infection and associated viruses in a birth cohort in the Philippines

**DOI:** 10.1186/s12879-022-07289-3

**Published:** 2022-03-30

**Authors:** Kanako Otani, Mayuko Saito, Michiko Okamoto, Raita Tamaki, Mariko Saito-Obata, Taro Kamigaki, Irene C. Lirio, Edelwisa Segubre-Mercado, Veronica Tallo, Socorro Lupisan, Hitoshi Oshitani

**Affiliations:** 1 Department of Virology, Tohoku University Graduate School of Medicine, 2-1 Seiryo-machi, Aoba-ku, Sendai, Miyagi 980-8575 Japan; 2 Japan International Cooperation Agency, Tokyo, Japan; 3 Department of Disease Surveillance and Epidemic Response, Ministry of Health, Nairobi, Kenya; 4 Research Institute for Tropical Medicine, Metro Manila, Philippines

**Keywords:** Lower respiratory tract infection, Birth cohort, Respiratory virus, Incidence, Philippines

## Abstract

**Background:**

Lower respiratory tract infection (LRTI) is an important cause of morbidity and mortality in infants and young children. However, the etiological role of viruses and the timing of developing LRTI are not well defined.

**Methods:**

We analyzed the data of a prospective cohort study in the Philippines as a birth cohort. We detected LRTI among children who visited healthcare facilities with respiratory symptom, and collected nasopharyngeal swabs for virus detection. We analyzed the incidence rates (IRs) and cumulative proportion of LRTI and severe LRTI by age group and each virus detected.

**Results:**

A total of 350 LRTI episodes were observed from 473 child-years yielded from 419 children. The IRs of LRTI were 70.8, 70.7, and 80.8 per 100 child-years for 0–5, 6–11, and 12–23 months of age, respectively. By 12 months of age, 45% of children developed LRTI at least once. Rhinovirus and respiratory syncytial virus were the most frequently detected viruses in all age groups. However, the IRs of influenza virus were low especially at 0–5 months of age.

**Conclusions:**

We identified various patterns of age-specific IRs of LRTI and severe LRTI for different viruses, which should be considered to establish more effective interventions including vaccinations.

**Supplementary Information:**

The online version contains supplementary material available at 10.1186/s12879-022-07289-3.

## Background

Lower respiratory tract infection (LRTI) is a leading cause of hospital admissions and mortality for children aged < 5 years worldwide [1]. It is estimated that more than 650,000 children in this age group died due to LRTI in 2016, and most of them occurred in low- and middle-income countries (LMICs) [2]. Bacterial pathogens such as *Streptococcus pneumoniae* and *Hemophilus influenzae* type b (Hib) have been shown to be a major cause of LRTI deaths in children [3, 4]. However, more recent studies have identified that respiratory viruses such as respiratory syncytial virus (RSV) [5] and influenza virus (IFV) [6] have a significant global disease burden among children, including a mortality impact, especially in LMICs.

Almost all children experience at least one episode of RSV infection by age 2 years [7], and infants < 3 months of age had the highest incidence of severe RSV infections [8]. In contrast, the incidence of severe IFV infections is higher in infants aged 6–11 months compare to those aged < 6 months [9]. It is important to understand the age-specific incidence of severe illnesses associated with different viruses to develop more effective strategies for interventions, including vaccinations. Effective vaccines for IFV are available, and the World Health Organization (WHO) strongly recommends pregnant women to be vaccinated to protect both the mothers and the infants [10]. There are still no available vaccines for RSV, but there are several promising vaccine candidates [11]. Both pregnant women and infants are considered to be a target group for RSV vaccination [12]. If infants aged < 6 months have a higher disease burden of RSV infections, maternal vaccination might be more important to minimize the impact of RSV. Although there are some available data on the incidences of virus-specific LRTI from birth cohort studies [8, 13], data are still not sufficient, especially in LMICs.

We have conducted a pediatric cohort study for children aged < 5 years in Biliran Island in the Philippines. We previously reported detailed incidence rates (IRs) and risk factors for RSV-associated LRTI (RSV-LRTI) using the cohort data [14]. In the present study, we analyzed the same cohort data as a birth cohort to identify the age-specific incidence of LRTI by different viruses. In addition to RSV, we also included IFV, adenovirus (AdV), rhinovirus (RV), enterovirus (EV), human metapneumovirus (MPV), and parainfluenza virus (PIV) in the analysis.

## Methods

### Study population

In the original cohort study, we followed children aged < 5 years who lived in two communities in Biliran Island from March 2014 to June 2016. Biliran Province consists of the main island (Biliran Island, 556 km^2^) and small islands with a total population of about 172,000 [15]. In Biliran Province, the main industry is agriculture and fishery, and socioeconomic status (SES) is generally low [16]. Pneumococcal-conjugate vaccines (PCV) and Hib conjugate vaccines were introduced in 2015 and 2013, respectively.

The study design of the original cohort study was described elsewhere [17, 18]. Briefly, children aged < 5 years were identified by a pre-study census that was conducted by visiting all households in the study areas. Pregnant women were identified at primary healthcare facilities or during the household visit, and newborn infants were also enrolled in the cohort throughout the study period. Demographic and socioeconomic information was obtained using a structured questionnaire at enrollment and the SES score was assessed as described elsewhere [19]. Study nurses visited each household every two weeks to check if cohort children had any respiratory symptoms and recommended the caregivers to take their children to healthcare facilities when children had difficulty breathing or chest indrawing.

In this study, we included only birth cohort children who were enrolled within 28 days after the birth in the analysis. They were followed up to 2 years of age or until the end of the original cohort study.

### Identification, classification, and case count of LRTI episodes

We included children who visited primary healthcare facilities or the outpatient clinic of the Biliran Provincial Hospital (BPH) with cough, coryza, or difficulty breathing, and those admitted to the BPH due to severe pneumonia based on the criteria of the Integrated Management of Childhood Illness (IMCI) [20, 21]. The BPH is the sole hospital providing secondary healthcare in the island. The study nurses or physician conducted physical examinations, including checking the presence of chest indrawing and measuring the respiratory rate and percutaneous oxygen saturation (SpO_2_) using a pulse oximeter (PalmSAT 2800; Nonin Medical Plymouth, MN). Illness history of cough, difficulty breathing, and danger signs (inability to feed, sleeping most of the time, or difficult to awake) were also obtained from the caregivers.

We defined and classified LRTI based on the definitions proposed by the WHO experts group [12] with some modifications. An episode of LRTI was defined when a child had cough or difficulty breathing and fast breathing (≥ 60 breaths per minute for children aged < 2 months, ≥ 50 breaths per min for children aged 2–11 months, ≥ 40 breaths per minute for children aged 12–23 months) or SpO_2_ < 95%. As children aged < 2 months were not included in the original definitions, the respiratory rate of ≥ 60 breaths per minute was used as fast breathing for this age group [22]. In the original definitions, severe LRTI and very severe LRTI were defined as LRTI with chest indrawing or SpO_2_ < 93% and LRTI with SpO_2_ < 90% or at least one of the danger signs (inability to feed, failure to respond, and unconsciousness), respectively. In this analysis, severe LRTI and very severe LRTI were combined as severe LRTI. Sleeping most of the time or difficult to awake was used to substitute failure to respond or unconsciousness in the original definitions. If an LRTI episodes did not meet the severe LRTI definitions, it was classified as “non-severe LRTI”. If SpO_2_ was measured after starting the oxygen treatment, the SpO_2_ value was considered as < 95%. If the severity was not determined due to incomplete SpO_2_ data, such a case was classified as “undefined LRTI”. Total LRTI included non-severe LRTI, severe LRTI, and undefined LRTI. An episode occurred after at least seven days without any respiratory symptoms was regarded as a new episode. If a child visited the healthcare facilities more than once during one episode, the most severe category of LRTI was used in the analysis.

### Laboratory diagnosis for virus detection

Nasopharyngeal swabs (NPS) were collected from cohort children who visited healthcare facilities. Conventional polymerase chain reaction (PCR) was performed to detect AdV [23], RV [24], EV [24] and PIV (PIV-1, PIV-2, PIV-3 and PIV-4) [25], and real-time PCR was used to detect IFV (IFV-A, including A(H1N1)pdm and A(H3N2), and IFV-B) [26]. Multiplex Real-time PCR was performed to detect RSV [27, 28] and MPV [29]. Positive samples were further classified into species or subgroups using Sanger sequencing for RV (RV-A, RV-B and RV-C) [24] and EV (EV-A, EV-B, EV-C and EV-D) [24], and PCR for RSV (RSV-A and RSV-B) [30, 31]. The details of the procedure were described elsewhere [17]. When multiple NPS samples were collected for one LRTI episode, all detected viruses were included in the analysis. Even if the child visited multiple health facilities, the laboratory test results were integrated using unique identification code of the study.

### Data analysis

Baseline characteristics were compared between age of follow-up using chi-square test. Incidence rates were calculated as the sum of episodes divided by the sum of the child-years observed in each age group. Poisson 95% confidential intervals (95% CIs) for IRs were computed. Incidence rate ratios (IRRs) were calculated by comparing the IRs between non-severe LRTI and severe LRTI and 95% CIs for IRRs were obtained using two-sided exact significance test. Cumulative proportions of children who developed LRTI were estimated using Kaplan–Meier survival analysis by the first and subsequent LRTI separately, and by detected viruses. When multiple viruses were detected in one LRTI episode, these LRTIs were included for each virus-specific survival curve. Stata version 15 (StataCorp, College Station, TX) was used for data analysis.

### Ethics approval

Informed consent was obtained from the guardians of all participants at enrollment. The study protocol was approved by the Ethics Committee of Tohoku University Graduate School of Medicine, Japan, and the Institutional Review Board of the Research Institute for Tropical Medicine (RITM), Philippines. All methods were performed in accordance with the relevant guidelines and regulations.

## Results

### Characteristics of cohort children

**Fig. 1:**
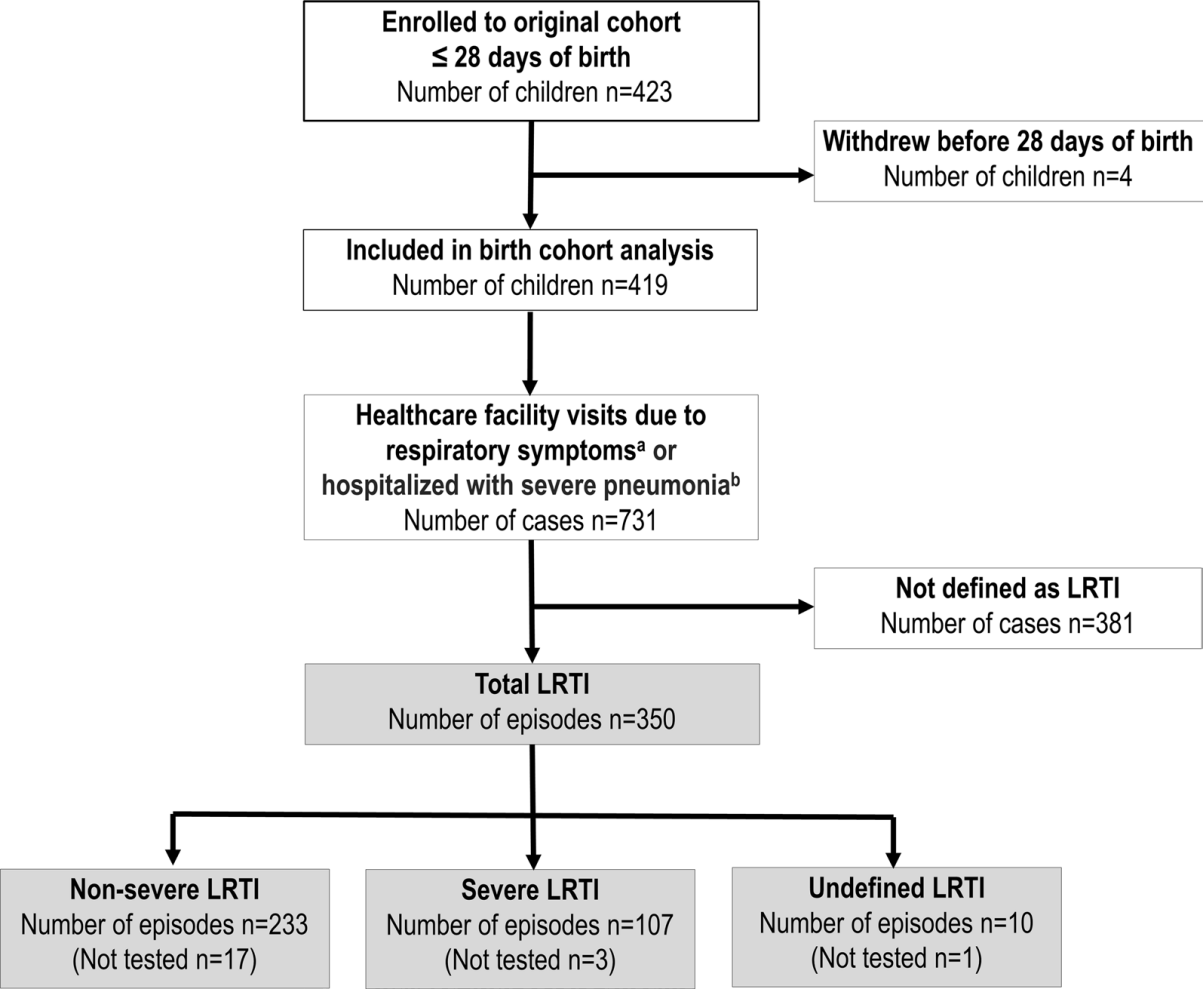
Schematic representation of the study participants and LRTI episodes detected in the birth cohort in Biliran, Philippines, from March 2014 to June 2016. ^a^Respiratory symptoms included cough, difficulty breathing or coryza. ^b^Diagnosis of severe pneumonia was based on the criteria of the Integrated Management of Childhood Illness (IMCI). Ten LRTI episodes were classified as undefined LRTI because SpO_2_ data were missing (n = 2) or it was not possible to assess as its measurement was performed after starting oxygen treatment (n = 8). Abbreviation: LRTI, lower respiratory tract infection; SpO_2_, percutaneous oxygen saturation. Almost all children in the study area were participated the study, though approximately 10 children refused to participate. Within 419 children, 183 children were followed until 1 year of age and 64 children were followed until 2 years of age, whereas 84 children were not followed until 6 months of age

A total of 419 children, after excluding four children who withdrew from the study before age 28 days, were included in the birth cohort analysis (Fig. 1), yielding 473 child-years of follow-up. The median age at enrollment was 14 days. In total, 183 children were followed until 1 year of age and 64 children were followed until 2 years of age, whereas 84 children were not followed until 6 months of age. The median follow-up age was 14.1 months (Interquartile range: 7.4–20.9). When we stratified the children according to their length of the follow-up, there was no significant difference in those characteristics among the groups except month of born. The baseline characteristics of participants by age of follow-up are shown in Additional file 3: Table S1. More than half of the children were from households with low SES score (< 30 indicating extreme poverty). The coverage of the PCV and Hib conjugate vaccine was 18.6% (46/247) and 58.3% (144/247), respectively, among children who were followed at least until 1 year of age.

### Classification of LRTI

Among 419 cohort children, 350 LRTI episodes were identified (Fig. 1). Of those, 13.1% (46/350) were classified as LRTI by the SpO_2_ value only. Of 350 episodes, 233 and 107 episodes were classified as non-severe LRTI and severe LRTI, respectively. For 10 LRTI episodes, the severity of LRTI was not classified (undefined LRTI).

### Incidence rates of total LRTI and severe LRTI

The overall IRs of total LRTI and severe LRTI were 74.0 (CI 66.4–82.2) and 22.6 (CI 18.5–27.3) per 100 child-years, respectively (Table 1). There were no differences in IRs of total LRTI between age groups (0–5 months: 70.8 (CI 58.9–84.4), 6–11 months: 70.7 (CI 57.7–85.7) and 12–23 months: 80.8 (CI 67.2–96.5)). The IR of severe LRTI for 0–5 months of age (25.7 per 100 child-year) was higher than that at 6–11 months (24.7 per 100 child-year) and 12–23 months of age (17.1 per 100 child-year), although it was not statistically significant (p = 0.866 and p = 0.096, respectively). The IRR of severe LRTI compared with non-severe LRTI was 0.58 (CI 0.40–0.86), 0.57 (CI 0.37–0.87) and 0.28 (CI 0.17–0.44) for 0–5 months, 6–11 months and 12–23 months of age, respectively.

**Table 1:** Incidence rates of LRTIs per 100 child-years and IRRs for severe LRTI compared to non-severe LRTI in Biliran, Philippines, from March 2014 to June 2016

	0–5 months			6–11 months			First year of life (0–11 months)			Second year of life (12–23 months)			Total		
LRTI classification	n	IR [95% CI]	IRR [95% CI]	n	IR [95% CI]	IRR [95% CI]	n	IR [95% CI]	IRR [95% CI]	n	IR [95% CI]	IRR [95% CI]	n	IR [95% CI]	IRR [95% CI]
Non-severe	77	44.0 [34.7–54.9]	1	63	43.2 [33.2–55.3]	1	140	43.6 [36.7–51.5]	1	93	61.1 [49.3–74.9]	1	233	49.3 [43.1–56.0]	1
Severe	45	25.7 [18.7–34.4]	0.58 [0.40–0.86]	36	24.7 [17.3–34.2]	0.57 [0.37–0.87]	81	25.2 [20.0–31.4]	0.58 [0.43–0.77]	26	17.1 [11.2–25.0]	0.28 [0.17–0.44]	107	22.6 [18.5–27.3]	0.46 [0.36–0.58]
Undefined	2	–	–	4	–	–	6	–	–	4	–	–	10	–	–
Total	124	70.8 [58.9–84.4]	–	103	70.7 [57.7–85.7]	–	227	70.7 [61.8–80.6]	–	123	80.8 [67.2–96.5]	–	350	74.0 [66.4–82.2]	–

Total LTRTI included non-severe LRTI, sever LRTI and undefined LRTI. Observed child-years were 175.2, 145.7, and 152.2 at age 0–5, 6–11, and 12–23 months, respectively

LRTI, lower respiratory tract infection; IR, incidence rate; IRR, incidence rate ratio; 95% CI, 95% confidence interval

### Cumulative proportion of children who developed LRTI

**Fig. 2:**
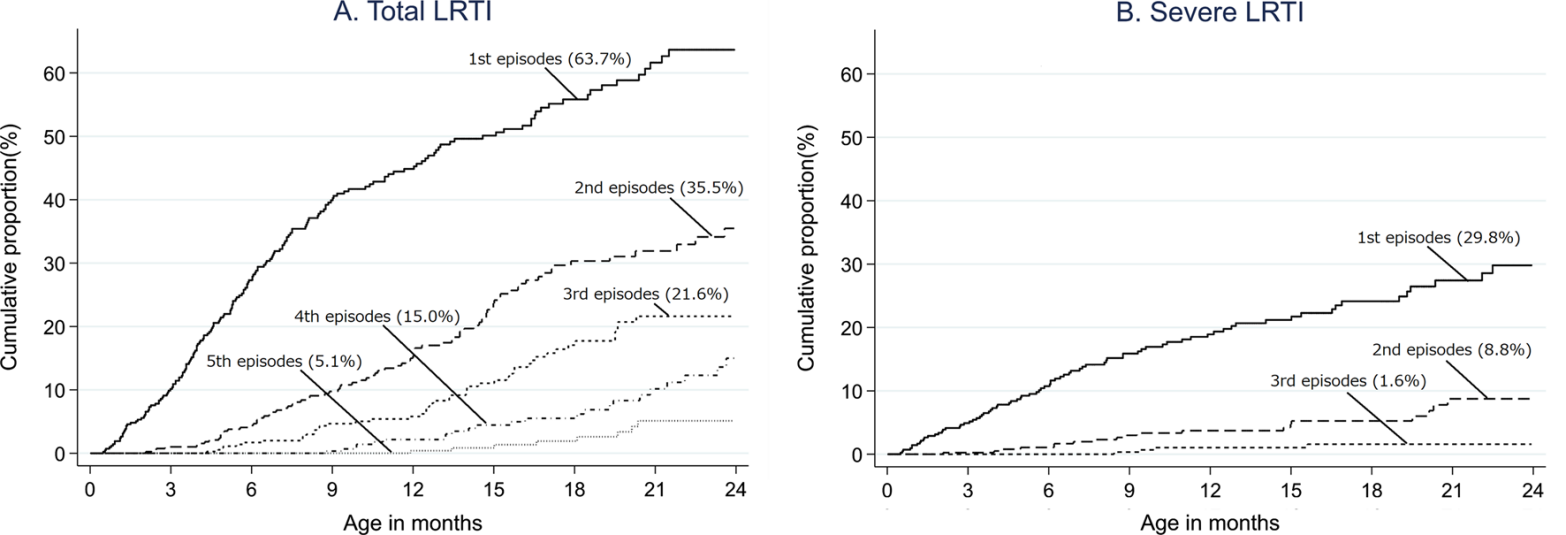
Cumulative proportion of children who developed first and subsequent LRTI. **A** Survival curves show cumulative proportions (%) of children who developed the first through fifth total LRTI episodes, including “non-severe LRTI”, “severe LRTI” and “undefined LRTI”. Although four children developed LRTI six times, the sixth episode is not shown in the figure. **B** Curves show the cumulative proportion (%) of children who developed "severe LRTI". Although two children developed severe LRTI four times and one developed it five times, the fourth and fifth episodes are not shown in the figure). Percentages (shown in brackets) show the cumulative proportion at 24 months of age. Abbreviation: LRTI, lower respiratory tract infection

The cumulative proportion of children who developed at least one LRTI was 44.9% and 63.7% at 12 and 24 months of age, respectively (Fig. 2A). The level of increase in the proportion of the first LRTI decelerated after 9 months. By 24 months, 35.5% of children developed second LRTI. There were 21.6% of children who experienced three or more LRTIs by 24 months. The cumulative proportion of children who developed severe LRTI was 18.9% and 29.8% by 12 and 24 months, respectively (Fig. 2B). The proportion of children who developed second severe LRTI by 24 months was estimated at 8.8%.

### Distribution of viruses detected in children with LRTI

Among 350 LRTI episodes, NPS samples were collected from 329 episodes (94.0%). Of those, at least one virus was detected from 228 episodes (69.3%). Among seven viruses, RV was the most frequently detected virus (87/329, 26.4%) followed by RSV (56/329, 17.0%) (Table 2), whereas IFV was detected in only 5.8% of LRTI episodes. When we compare the detection rates by severity of LRTIs, RSV was more frequently detected in severe LRTI (23/104, 22.1%) than in non-severe LRTI (30/216, 13.9%) and PIV was detected more in non-severe LRTI (22/216, 10.2%) than in severe LRTI (6/104, 5.8%). However, the differences did not reach the statistical significance (p = 0.064, p = 0.190, respectively). Two viruses were detected from 16 episodes, and co-detection of RSV and RV was most frequently observed (8/16, Additional file 4: Table S2). The epidemic curves of each virus in the original cohort study of children aged < 5 years are shown in Additional file 1: Figure S1.

**Table 2:** Detected viruses and subtypes or species from cases with LRTI in Biliran, Philippines, from March 2014 to June 2016

Detected virus	Non-severe LRTI	Severe LRTI	Undefined LRTI	Total
AdV	3 (1.4%)	0 (0.0%)	0 (0.0%)	3 (0.9%)
RV	59 (27.3%)	26 (25.0%)	2 (22.2%)	87 (26.4%)
RV-A	33	13	2	48
RV-B	5	0	0	5
RV-C	21	13	0	34
EV	6 (2.8%)	4 (3.9%)	0 (0.0%)	10 (3.0%)
EV-A	1	0	0	1
EV-B	3	1	0	4
EV-C	1	0	0	1
EV-D (D68)	1	3	0	4
RSV	30 (13.9%)	23 (22.1%)	3 (33.3%)	56 (17.0%)
RSV-A	3	9	1	13
RSV-B	27	12	2	41
RSV-untyped	0	2	0	2
MPV	6 (2.8%)	3 (2.9%)	0 (0.0%)	9 (2.7%)
PIV	22 (10.2%)	6 (5.8%)	0 (0.0%)	28 (8.5%)
PIV-1	3	3	0	6
PIV-2	1	0	0	1
PIV-3	17	3	0	20
PIV-4	1	0	0	1
IFV	11 (5.1%)	7 (6.7%)	1 (11.1%)	19 (5.8%)
IFV-A	10	6	1	17
IFV-B	1	1	0	2
Co-detection	11 (5.1%)	4 (3.9%)	1 (11.1%)	16 (4.9%)
Negative	68 (31.5%)	31 (29.8%)	2 (22.2%)	101 (30.7%)
Sub-total	216 (100.0%)	104 (100.0%)	9 (100.0%)	329 (100.0%)
Not tested	17	3	9	21
Total	233	107	10	350

LRTI, lower respiratory tract infection; AdV, Adenovirus; RV, Rhinovirus; EV, Enterovirus; RSV, Respiratory syncytial virus; MPV, Human metapneumovirus; PIV, Parainfluenza virus; IFV, Influenza virus

### Cumulative proportion of children who developed virus-specific LRTI

**Fig. 3:**
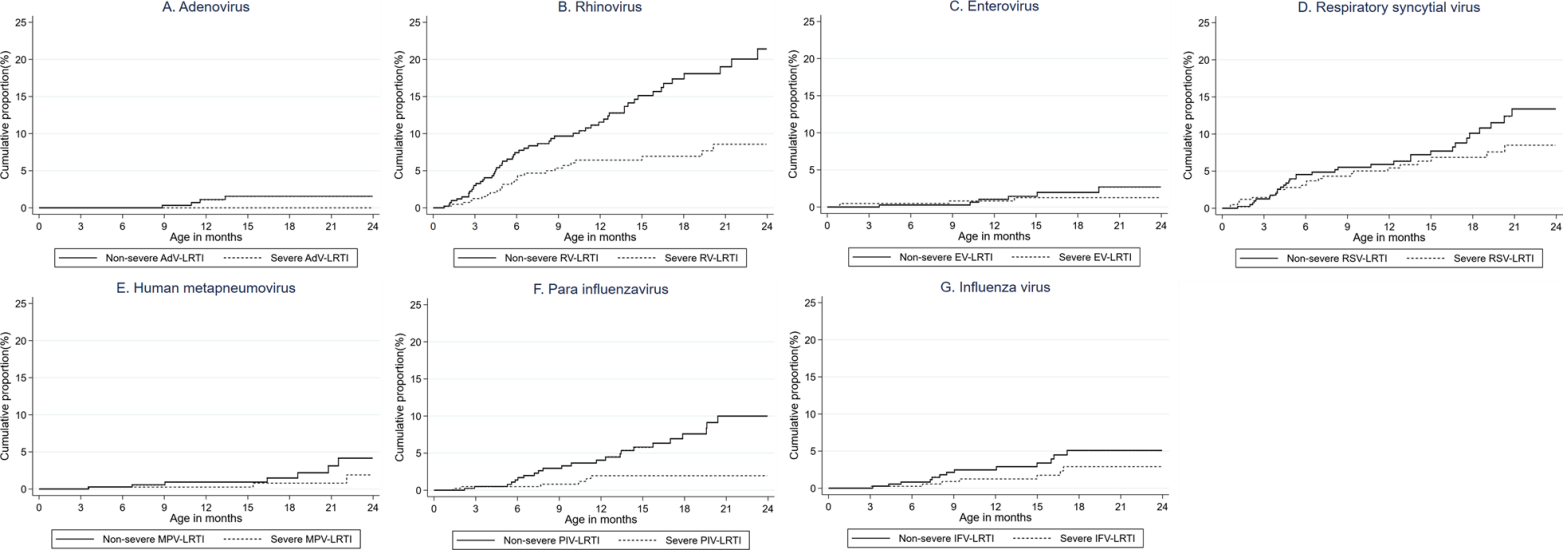
Cumulative proportion of children who developed non-severe and severe virus-specific LRTI by virus. The number of episodes (non-severe LRTI, severe LRTI) used for the analysis were AdV (3, 0), RV (59, 26), EV (6, 4), RSV (30, 23), MPV (6, 3), PIV (22, 6) and IFV (11, 7), respectively. Abbreviation: LRTI, lower respiratory tract infection; AdV, adenovirus; RV, rhinovirus; EV, enterovirus; RSV, respiratory syncytial virus; MPV, human metapneumovirus; PIV, para influenzavirus; IFV, influenza virus

Figure 3 shows the cumulative proportion of virus-specific LRTI by severity. At the age of 24 months, the cumulative proportion of non-severe and severe LRTI was 21.4% and 8.6% for RV-associated LRTI (RV-LRTI). The gap between non-severe and sever RV-LRTI became wider after 3 months of age. On the other hand, the gap was not observed until 15 months of age for RSV-LRTI. The cumulative proportion of non-severe RSV-LRTI and severe RSV-LRTI at 24 month of age was 13.4% and 8.5%, respectively.

The cumulative proportion of RV-LRTI and RSV-LRTI increased from < 1 month of age (Additional file 2: Figure S2, Fig. 3B and D). In contrast, the proportion of MPV-associated LRTI (MPV-LRTI) and IFV-associated LRTI (IFV-LRTI) did not increase until the first 3 months of age (Additional file 2: Figure S2, Fig. 3E and G).

### Incidence of virus-specific LRTI by age group

When the IRs of the virus-specific LRTIs were stratified by age group, the IRs of RV- and RSV-LRTIs for 0–5 months of age were higher than those for 6–11 and 12–23 months (Table 3). In contrast, the IRs of EV-associated (EV-), MPV-, PIV-associated (PIV-) and IFV-LRTIs were higher in the older age group. However, these differences were not statistically significant (all p ≥ 0.05). When the virus-specific LRTIs were stratified by age group and severity, the IRR of severe RSV-LRTI to non-severe RSV-LRTI was higher for 6–11 months of age (IRR: 2.33, CI 0.53–13.98) than for0–5 months (IRR: 0.73, CI 0.30–1.71) and 12–23 months of age (IRR: 0.42, CI 0.11–1.27) without statistical significance (Table 3).

**Table 3:** Incidence rates of virus-specific LRTIs per 100 child-years and IRRs for severe LRTI compared to non-severe LRTI in Biliran, Philippines, from March 2014 to June 2016

Detected virus	LRTI classification	0–5 months					6–11 months					12–23 months				
		n	IR	[95%CI]	IRR	[95%CI]	n	IR	[95%CI]	IRR	[95%CI]	n	IR	[95%CI]	IRR	[95%CI]
AdV	Non-severe	0	0.0	[0.0–2.1]	1		2	1.4	[0.2–5.0]	1		1	0.7	[0.0–3.7]	1	
	Severe	0	0.0	[0.0–2.1]	0.00	[0.00–0.00]	0	0.0	[0.0–2.5]	0.00	[0.00–5.32]	0	0.0	[0.0–2.4]	0.00	[0.00–39.00]
	Total	0	0.0	[0.0–2.1]	–	–	2	1.4	[0.2–5.0]	–	–	1	0.7	[0.0–3.7]	–	–
RV	Non-severe	24	13.7	[8.8–20.4]	1		13	8.9	[4.8–15.3]	1		22	14.5	[9.1–21.9]	1	
	Severe	15	8.6	[4.8–14.1]	0.63	[0.30–1.24]	7	4.8	[1.9–9.9]	0.54	[0.18–1.45]	4	2.6	[0.7–6.7]	0.18	[0.05–0.54]
	Total	39	22.3	[15.8–30.4]	–	–	21	14.4	[8.9–22.0]	-	-	27	17.7	[11.7–25.8]	–	–
EV	Non-severe	1	0.6	[0.0–3.2]	1		2	1.4	[0.2–5.0]	1		3	2.0	[0.4–5.8]	1	
	Severe	2	1.1	[0.1–4.1]	2.00	[0.10–117.99]	1	0.7	[0.0–0.0]	0.50	[0.01–9.60]	1	0.7	[0.0–3.7]	0.33	[0.01–4.15]
	Total	3	1.7	[0.4–5.0]	–	–	3	2.1	[0.4–6.0]	-	-	4	2.6	[0.7–6.7]	–	–
RSV	Non-severe	15	8.6	[4.8–14.1]	1		3	2.1	[0.4–6.0]	1		12	7.9	[4.1–13.8]	1	
	Severe	11	6.3	[3.1–11.2]	0.73	[0.30–1.71]	7	4.8	[1.9–9.9]	2.33	[0.53–13.98]	5	3.3	[1.1–7.7]	0.42	[0.11–1.27]
	Total	27	15.4	[10.2–22.4]	–	–	11	7.6	[3.8–13.5]	–	–	18	11.8	[7.0–18.7]	–	–
MPV	Non-severe	1	0.6	[0.0–3.2]	1		1	0.7	[0.0–3.8]	1		4	2.6	[0.7–6.7]	1	
	Severe	1	0.6	[0.0–3.2]	1.00	[0.01–78.50]	0	0.0	[0.0–2.5]	0.00	[0.00–39.00]	2	1.3	[0.2–4.7]	0.50	[0.05–3.49]
	Total	2	1.1	[0.1–4.1]	–	–	1	0.7	[0.0–3.8]	–	–	6	3.9	[1.5–8.6]	–	–
PIV	Non-severe	4	2.3	[0.6–5.8]	1		7	4.8	[1.9–9.9]	1		11	7.2	[3.6–12.9]	1	
	Severe	2	1.1	[0.1–4.1]	0.50	[0.05–3.49]	4	2.7	[0.7–7.0]	0.57	[0.12–2.25]	0	0.0	[0.0–2.4]	0.00	[0.00–0.40]
	Total	6	3.4	[1.37.5]	–	–	11	7.6	[3.8–13.5]	–	–	11	7.2	[3.6–12.9]	–	–
IFV	Non-severe	3	1.7	[0.4–5.0]	1		4	2.7	[0.7–7.0]	1		4	2.6	[0.7–6.7]	1	
	Severe	1	0.6	[0.0–3.2]	0.33	[0.01–4.15]	3	2.1	[0.4–6.0]	0.75	[0.11–4.43]	3	2.0	[0.4–5.8]	0.75	[0.11–4.43]
	Total	5	2.9	[0.9–6.7]	–	–	7	4.8	[1.9–9.9]	–	–	7	4.6	[1.9–9.5]	–	–
Co-detection	Non-severe	7	4.0	[1.6–8.2]	1		3	2.1	[0.4–6.0]	1		1	0.7	[0.0–3.7]	1	
	Severe	2	1.1	[0.1–4.1]	0.29	[0.03–1.50]	1	0.7	[0.0–3.8]	0.33	[0.01–4.15]	1	0.7	[0.0–3.7]	1.00	[0.01–78.50]
	Total	9	5.1	[2.4–9.8]	–	–	4	2.7	[0.8–7.0]	–	–	3	2.0	[0.4–5.8]	–	–
Negative	Non-severe	20	11.4	[7.0–17.6]	1		20	13.7	[8.4–21.2]	1		28	18.4	[12.2–26.6]	1	
	Severe	11	6.3	[3.1–11.2]	0.55	[0.24–1.20]	13	8.9	[4.8–15.3]	0.65	[0.30–1.37]	7	4.6	[1.8–9.5]	0.25	[0.09–0.59]
	Total	31	17.7	[12.0–25.1]	–	–	34	23.3	[16.2–32.6]	–	–	36	23.7	[16.6–32.8]	–	–
Total	Non-severe	75	42.8	[33.7–53.7]	1		55	37.8	[28.4–49.1]	1		86	56.5	[45.2–69.8]	1	
	Severe	45	25.7	[18.7–34.4]	0.61	[0.41–0.88]	36	24.7	[17.3–34.2]	0.65	[0.42–1.01]	23	15.1	[9.6–22.7]	0.27	[0.16–0.43]
	Total	122	69.6	[57.8–83.2]	–	–	94	64.5	[52.1–79.0]	–	–	113	74.3	[61.2–89.3]	–	–

Total LTRTI included non-severe LRTI, severe LRTI and undefined LRTI. Observed child-years were 175.2, 145.7, and 152.2 at age 0–5, 6–11, and 12–23 months, respectively. Episodes in which samples were not tested for virus detection were excluded (n = 21)

IR, incidence rate; IRR, incidence rate ratio; LRTI, lower respiratory tract infection; 95%CI, 95% confidence interval; AdV, Adenovirus; RV, Rhinovirus; EV, Enterovirus; RSV, Respiratory syncytial virus; MPV, Human metapneumovirus; PIV, Parainfluenza virus; IFV, Influenza virus

## Discussion

To our knowledge, this is the first study that applied the definition of LRTI with SpO_2_ measurement by the WHO Working Group [12] for major respiratory viruses. Our data demonstrated the high IR of LRTI for children aged < 2 years in rural areas of the Philippines (74.0 per 100 child-years). For children aged < 1 year, the IR of LRTI (70.7 per 100 child-years) was higher than that of the study in South Africa (51 per 100 child-years) [32]. Although most of the other studies used the definitions of pneumonia of the IMCI [32–34] to define LRTI, we used the definitions proposed by the WHO Working Group [12], which include the SpO_2_ value to define LRTI. This can be a reason for the high IRs of LRTI. However, only 13% of LRTI episodes in our study were defined by the SpO_2_ value. Other factors such as low vaccine coverage for PCV and Hib conjugate vaccine in our study population may also have contributed to the high IRs. However, we did not test blood or respiratory samples for bacterial pathogens. Blood exaction was not feasible in our study setting because most of the episodes were identified in outpatient setting and the bacteremia was rare even in the hospitalized children with severe pneumonia in our previous study [20]. In addition, etiological significance is limited in children with pneumonia [35, 36]. Even higher IR of LRTI in the first year of life was reported from other cohort studies in Bangladesh (88.3 and 76.0 per 100 child-years for 0–5 and 6–11 months of age, respectively) [34] and India (96.0 per 100 child-years for 0–11 months of age) [33]. The incidence of LRTI may vary even within the country. The national statistics of the Philippines showed that Eastern Visayas Region to which Biliran Province belongs had a much higher incidence of acute LRTI and pneumonia than the national average [37]. Since our study was conducted in two rural communities in a small island, the results may not be applicable for the whole Philippines. However, our previous study confirmed similar patterns of viral etiology in four hospitals in different regions of the Philippines including Metro Manila and Biliran Province [20]. Therefore, we believe that infants and young children in other parts of the country are likely to have similar patterns of viral infections.

We observed high IRs of LRTI in all age groups, 70.7– 80.8 per 100 child years in 0–5, 6–11 and 12–23 months of age. High IR of LRTI in children aged 12–23 months was due to the high IR of non-severe LRTI, and most of these LRTI were repeated LRTI. Other cohort studies have shown different patterns of age-specific IRs of LRTI. The study in Bangladesh showed higher IR at younger age groups and lower IRs in the older age group (88.3, 76.0 and 57.2 per 100 child-year in 0–5, 6–11 and 12–23 months) [34]. Lower IRs for 12–23 months of age compare to 0–11 months were also reported in other studies in South Africa (51 vs 25 per 100 child-year) and India (96 vs 52 per 100 child-year) [32, 33]. However, some other studies (37.0 vs 83.1 per 100 child-year) and Australia (shown in the figure) indicated even higher IRs for 12–23 of age months than 0–11 months [38, 39]. Such difference might be due to different incidences of causative agents. In general, IFV and PIV are more commonly identified from older children compared with RSV and RV [46]. The study in India that showed higher incidence of LRTI for 12–23 months of age had high positivity rates for IFV and PIV [38], whereas most of other studies including our study, had high positivity rates for RSV and RV.

At least one virus was detected in 69.3% of LRTI episodes, which indicate the importance of respiratory viruses in LRTI for children aged < 2 years. The most commonly identified virus was RV (26.4%) followed by RSV (17.0%). RSV has been recognized as the most important viral pathogens associated with hospitalized LRTI cases in infants and young children [5]. In our data, the highest IRs of RSV-LRTI and severe RSV-LRTI were observed in infants aged 0–5 months. A high IR of RSV associated pneumonia in this age group was reported from birth cohort study in Mali [40]. This age group is considered to be a prime target for RSV interventions including future RSV vaccinations [12]. However, we also observed a relatively high IR of RSV-LRTI in children aged 12–23 months, which was similar to our previous analysis of the same data set [14] and other studies [41, 42]. Although the IR of RSV-LRTI for 6–11 months of age was lower than those for 0–5 and 12–23 months of age, the IR of severe RSV-LRTI in this age group was higher than in 12–23 months, and IRR for severe LRTI compared to non-severe LRTI was even higher than that for 0–5 months of age. The same pattern was observed in the birth cohort study in Kenya [41]. When establishing the RSV vaccination strategy, it may be necessary to reduce the impact in older infants and young children.

In the present study, RV showed the highest IRs among detected viruses in all age groups. For 0–5 months of age, the IR of severe RV-LRTI was particularly high and incidence of RV-LRTI and severe RV-LRTI increased immediately after birth. However, the etiological importance of RV for LRTI in infants and young children is still controversial. A birth cohort study in Australia, which included healthy controls, showed that RV is more important than RSV as a cause of LRTI in infants < 1 year of age [43]. On the other hand, another birth cohort study in South Africa indicated that RV had limited attribution to LRTI [44]. A meta-analysis also showed a low attributable fraction of RV [45]. Other studies also showed that many of RV infections were mild or asymptomatic, although RV was the most commonly detected virus [46, 47]. Analysis of attributable fraction by comparing RV positive rates between symptomatic cases and healthy control may underestimate a real impact of RV since virus shedding period after symptomatic RV infections may be longer [48]. Further studies are necessary to define an exact impact of RV in infants and young children.

The overall positivity rates of other viruses, including AdV, EV, MPV, PIV, and IFV were low ranging from 0.9% (AdV) to 8.5% (PIV). It has been shown that seasonal IFV has high disease burden particularly in infants aged < 1 year [49]. Therefore, the WHO strongly recommends maternal IFV vaccination to protect both pregnant women and infants [50]. However, in our data, IFV was detected only in 5.8% of LRTI episodes and the IRs of IFV-LRTI were relatively low in all age groups, especially in infants aged < 6 months (2.9 per 100 child-years), with only one severe IFV-LRTI in this age group. Other cohort studies also indicated lower IRs of IFV among infants compare to those of RV and RSV [34, 43, 44]. Further studies are necessary to define the disease burden of seasonal IFV in infants and the effectiveness of maternal vaccination. The detection rate of PIV (8.5%) in LRTI episodes was higher than IFV (5.8%). This was mainly due to a relatively large outbreak of PIV-3 during the study period. Incidences of other viruses including AdV, EV, and MPV were low. Although AdV, especially AdV type 7 [51], and EV, especially EV-D68 [52] were shown to be associated with severe LRTI with some fatal episodes in our hospital-based studies in the Philippines, the present study suggested a relatively small contribution of these viruses to overall LRTI in the study population.

The study has some limitations. First, we conducted the study for only 2 years and 3 months by recruiting newborns throughout the study period. Therefore, the observed period for each age group varied (Additional file 1: Figure S1). For example, the observation period for 12–23 months only covered 2015 and 2016. As viruses detected in the study had some seasonality and annual fluctuation, the age and virus-specific IRs are affected by various observation periods among age groups. Second, we detected episodes with LRTI only at healthcare facilities. However, more than 90% of children in the study area sought care at healthcare facilities when they had pneumonia-like episode [16]. We believe that a majority of LRTI episodes were detected in the present study. Third, there was some delay in the recruitment of newborn babies for the study (median 14 days). We probably have missed some LRTI episodes that occurred before recruitment.

In conclusion, we identified various patterns of age-specific IRs of LRTI and severe LRTI for different viruses, which should be considered to establish more effective interventions including vaccinations.

## Supplementary Information

**Additional file 1: Figure S1.** Number of LRTI episodes and number of children (mean) stratified by age group in the birth cohort, and number of virus positive samples observed in the original cohort study (among children aged < 5 years) in Biliran, Philippines, from March 2014 to June 2016.

**Additional file 2: Figure S2.** Cumulative proportion of children who developed non-severe and severe virus-specific LRTI.

**Additional file 3: Table S1.** Characteristics of children in the birth cohort study by age at the end of follow-up in Biliran, Philippines, from March 2014 to June 2016.

**Additional file 4: Table S2.** Details of the detected viruses among cases with LRTI in which two viruses were detected in Biliran, Philippines, from March 2014 to June 2016.

## Data Availability

The datasets generated and analysed during the current study are not publicly available due to ongoing research and analysis, but are available from the corresponding author on reasonable request.

## Abbreviations

LRTI: Lower respiratory tract infection
AdV: Adenovirus
RV: Rhinovirus
EV: Enterovirus
RSV: Respiratory syncytial virus
MPV: Human metapneumovirus
PIV: Parainfluenza virus
IFV: Influenza virus
IRs: Incidence rates
IRRs: Incidence rate ratios
95%CIs: 95% Confidential intervals
SpO_2_: Percutaneous oxygen saturation
Hib: *Hemophilus influenzae* Type b
WHO: World Health Organization
SES: Socioeconomic status
PCV: Pneumococcal-conjugate vaccines
IMCI: Integrated Management of Childhood Illness
NPS: Nasopharyngeal swabs
