# Supplementary material for: Incidence of lower respiratory tract infection and associated viruses in a birth cohort in the Philippines
Source: BMC Infect Dis. 2022 Mar 30;22:313. doi: 10.1186/s12879-022-07289-3 (PMC8966153; doi:10.1186/s12879-022-07289-3)
Supplement: Supplementary file 1 — Additional file 1: Figure S1. Number of LRTI episodes and number of children (mean) stratified by age group in the birth cohort, and number of virus positive samples observed in the original cohort study (among children aged < 5 years) in Biliran, Philippines, from March 2014 to June 2016. [file 12879_2022_7289_MOESM1_ESM.pptx]

## Slide 1
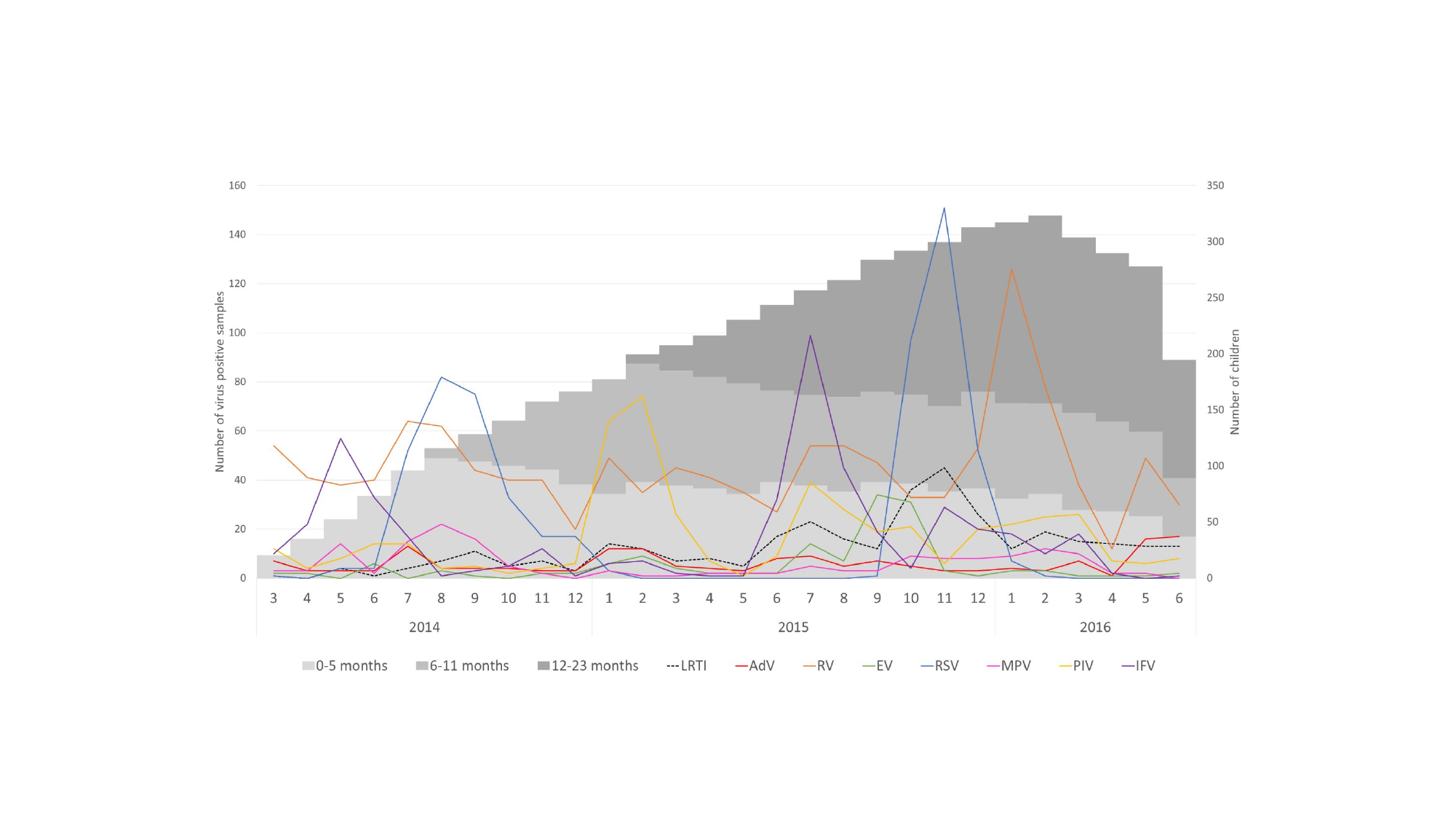

## Slide 2
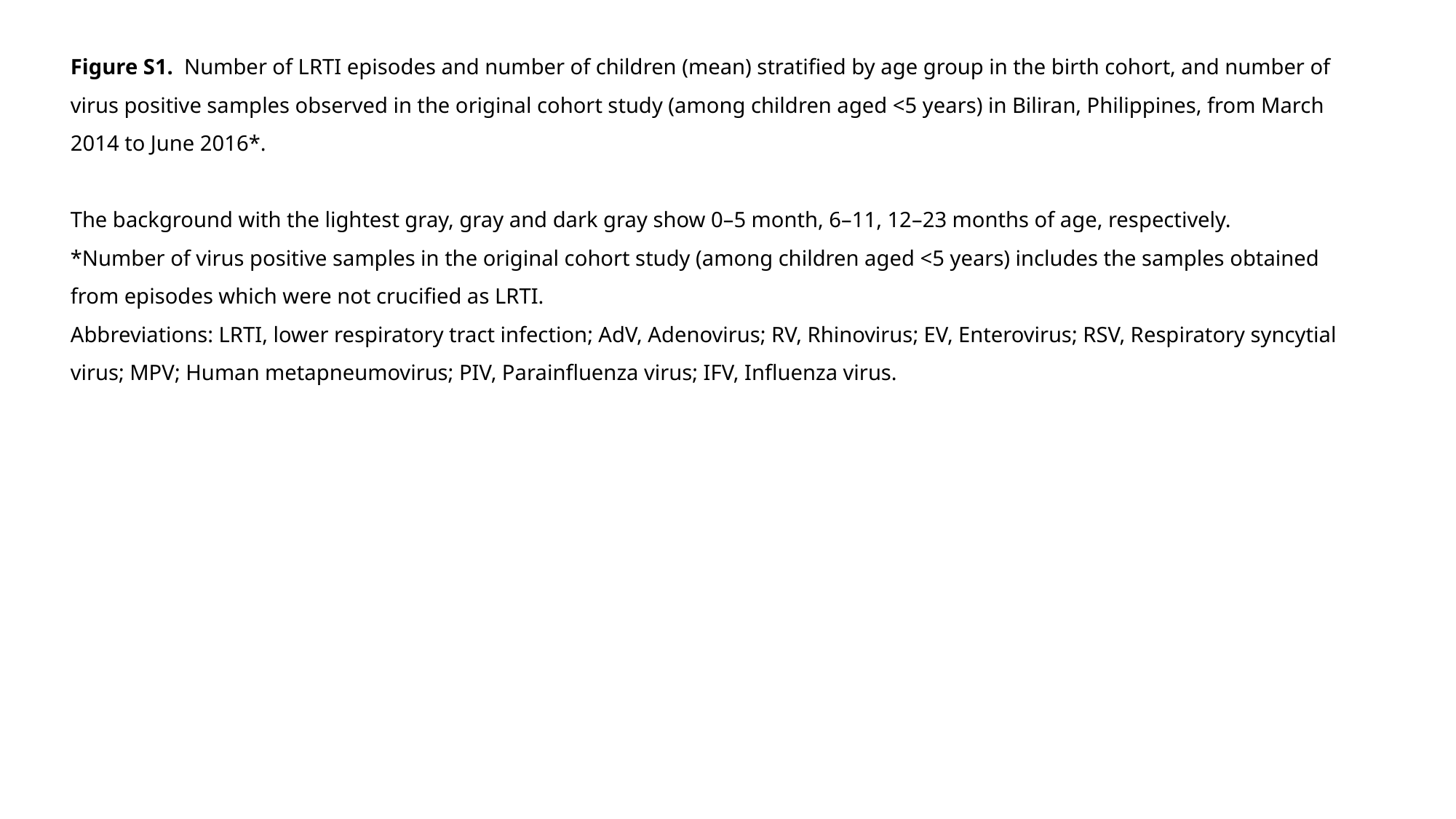

Figure S1. Number of LRTI episodes and number of children (mean) stratified by age group in the birth cohort, and number of virus positive samples observed in the original cohort study (among children aged <5 years) in Biliran, Philippines, from March 2014 to June 2016*.
The background with the lightest gray, gray and dark gray show 0–5 month, 6–11, 12–23 months of age, respectively.
*Number of virus positive samples in the original cohort study (among children aged <5 years) includes the samples obtained from episodes which were not crucified as LRTI.
Abbreviations: LRTI, lower respiratory tract infection; AdV, Adenovirus; RV, Rhinovirus; EV, Enterovirus; RSV, Respiratory syncytial virus; MPV; Human metapneumovirus; PIV, Parainfluenza virus; IFV, Influenza virus.
