# Supplementary material for: Incidence of lower respiratory tract infection and associated viruses in a birth cohort in the Philippines
Source: BMC Infect Dis. 2022 Mar 30;22:313. doi: 10.1186/s12879-022-07289-3 (PMC8966153; doi:10.1186/s12879-022-07289-3)
Supplement: Supplementary file 2 — Additional file 2: Figure S2. Cumulative proportion of children who developed non-severe and severe virus-specific LRTI. [file 12879_2022_7289_MOESM2_ESM.pptx]

## Slide 1
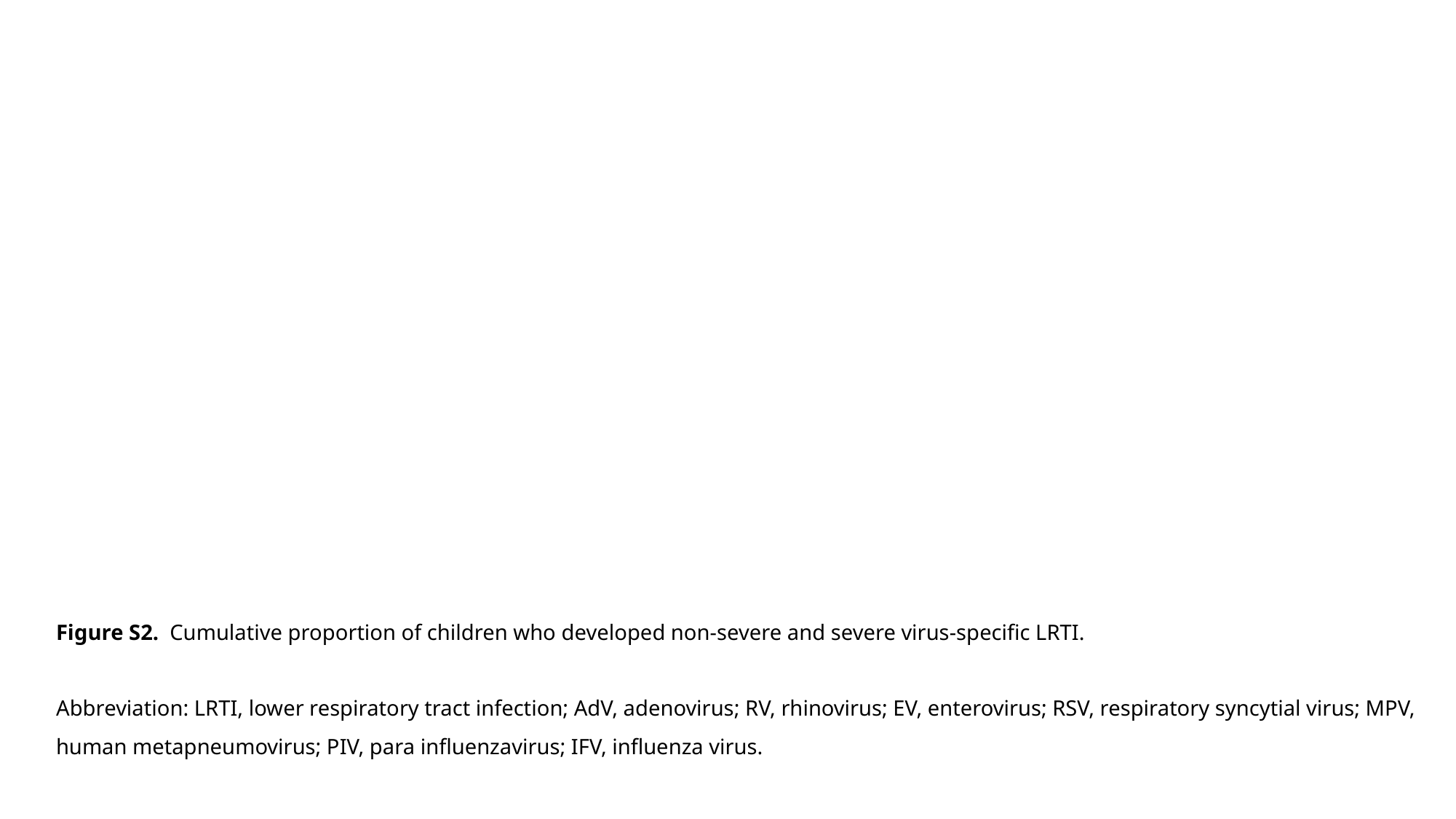

Figure S2. Cumulative proportion of children who developed non-severe and severe virus-specific LRTI.
Abbreviation: LRTI, lower respiratory tract infection; AdV, adenovirus; RV, rhinovirus; EV, enterovirus; RSV, respiratory syncytial virus; MPV, human metapneumovirus; PIV, para influenzavirus; IFV, influenza virus.
