# Supplementary material for: Incidence of lower respiratory tract infection and associated viruses in a birth cohort in the Philippines
Source: BMC Infect Dis. 2022 Mar 30;22:313. doi: 10.1186/s12879-022-07289-3 (PMC8966153; doi:10.1186/s12879-022-07289-3)
Supplement: Supplementary file 3 — Additional file 3: Table S1. Characteristics of children in the birth cohort study by age at the end of follow-up in Biliran, Philippines, from March 2014 to June 2016. [file 12879_2022_7289_MOESM3_ESM.docx]

|  |  |  | Age at the end of follow-up | | |  |
| --- | --- | --- | --- | --- | --- | --- |
|  |  | Total | <6 months | 6–11 months | ≥12 months |  |
| Characteristics |  | n=419 | n=84^a^ | n=88 | n=247 | p-value |
| Sex | Male | 212 (50.6) | 43 (51.2) | 40 (45.5) | 129 (52.2) |  |
|  | Female | 207 (49.4) | 41 (48.8) | 48 (54.6) | 118 (47.8) | 0.547 |
| Place of Living | Kawayan | 207 (49.4) | 34 (40.5) | 44 (50.0) | 129 (52.2) |  |
|  | Caibiran | 212 (50.6) | 50 (59.5) | 44 (50.0) | 118 (47.8) | 0.176 |
| Month of born | Jan-Mar | 115 (27.5) | 41 (48.8) | 2 (2.3) | 72 (29.2) |  |
|  | Apr-Jun | 135 (32.2) | 25 (29.8) | 12 (13.6) | 98 (39.7) |  |
|  | Jul-Sep | 92 (22.0) | 8 (9.5) | 43 (48.9) | 41 (16.6) |  |
|  | Oct-Dec | 77 (18.4) | 10 (11.9) | 31 (35.2) | 36 (14.6) | <0.001 |
| Birth weight | ≥2500g | 333 (86.7) | 70 (89.7) | 67 (83.8) | 196 (86.7) |  |
|  | <2500g | 51 (13.3) | 8 (10.3) | 13 (16.3) | 30 (13.3) | 0.540 |
| Preterm | No | 413 (99.3) | 82 (98.8) | 87 (100.0) | 244 (99.2) |  |
|  | Yes | 3 (0.7) | 1 (1.2) | 0 (0.0) | 2 (0.8) | 0.627 |
| Exclusive  breastfeeding | ≥150days | 207 (59.1) | 27 (49.1) | 36 (54.6) | 144 (62.9) |  |
|  | <150days | 119 (34.0) | 21 (38.2) | 25 (39.9) | 73 (31.9) |  |
|  | Never | 24 (6.9) | 7 (12.7) | 5 (7.6) | 12 (5.2) | 0.179 |
| Birth order | First | 84 (20.1) | 17 (20.2) | 19 (21.6) | 48 (19.4) |  |
|  | Second | 107 (25.5) | 24 (28.6) | 25 (28.4) | 58 (23.5) |  |
|  | Third | 228 (54.4) | 43 (51.2) | 44 (50.0) | 141 (57.1) | 0.754 |
| Living with  biological mother | Yes | 412 (98.3) | 83 (98.8) | 87 (98.9) | 242 (98.0) |  |
|  | No | 7 (1.7) | 1 (1.2) | 1 (1.1) | 5 (2.0) | 0.795 |
| Atopy history of  family | No | 291 (69.5) | 60 (71.4) | 60 (68.2) | 171 (69.2) |  |
|  | Yes | 128 (30.6) | 24 (28.6) | 28 (31.8) | 76 (30.8) | 0.893 |
| Number of family  members | <7 | 206 (49.2) | 37 (44.1) | 43 (48.9) | 126 (51.0) |  |
|  | ≥7 | 213 (50.8) | 47 (56.0) | 45 (51.1) | 121 (49.0) | 0.543 |
| HH crowding^b^ | <2 | 89 (21.2) | 16 (19.1) | 19 (21.6) | 54 (21.9) |  |
|  | ≥2 | 330 (78.8) | 68 (81.0) | 69 (78.4) | 193 (78.1) | 0.859 |
| Caregiver's  smoking status | No | 404 (96.4) | 80 (95.2) | 86 (97.7) | 238 (96.4) |  |
|  | Yes | 15 (3.6) | 4 (4.8) | 2 (2.3) | 9 (3.6) | 0.678 |
| Having smoker in  the same HH | No | 155 (37.0) | 31 (36.9) | 37 (42.1) | 87 (35.2) |  |
|  | Yes | 264 (63.0) | 53 (63.1) | 51 (58.0) | 160 (64.8) | 0.523 |
| Education level of  mother | < 6 years | 95 (22.7) | 21 (25.0) | 17 (19.3) | 57 (23.1) |  |
|  | 6–11 years | 243 (58.0) | 46 (54.8) | 53 (60.2) | 144 (58.3) |  |
|  | ≥12 years | 81 (19.3) | 17 (20.2) | 18 (20.5) | 46 (18.6) | 0.903 |
| Education level of  father | < 6 years | 152 (42.7) | 33 (50.0) | 24 (34.8) | 95 (43.0) |  |
|  | 6–11 years | 146 (41.0) | 26 (39.4) | 32 (46.4) | 88 (39.8) |  |
|  | ≥12 years | 58 (16.3) | 7 (10.6) | 13 (18.8) | 38 (17.2) | 0.387 |
| SES score | ≥30 | 202 (48.2) | 40 (47.6) | 48 (54.6) | 114 (46.2) |  |
|  | <30 | 217 (51.8) | 44 (52.4) | 40 (45.5) | 133 (53.9) | 0.398 |

**Table S1.** Characteristics of children in the birth cohort study by age at the end of follow-up in Biliran, Philippines, from March 2014 to June 2016.

^a^ Nineteen out of 84 children were aged <6 months at the end of the study period.

^b^HH crowding was calculated as number of family members divided by number of rooms.

Abbreviations: HH, household; SES, Socioeconomic status.

The distribution of each characteristics was compared among three groups of participants categorized by the duration of the follow-up using chi-square test. Missing data of birth weight (n=35), preterm (n=3), exclusive breastfeeding (n=69), and education level of father (n=63) were excluded from the analysis.
