# Supplementary material for: Incidence of lower respiratory tract infection and associated viruses in a birth cohort in the Philippines
Source: BMC Infect Dis. 2022 Mar 30;22:313. doi: 10.1186/s12879-022-07289-3 (PMC8966153; doi:10.1186/s12879-022-07289-3)
Supplement: Supplementary file 4 — Additional file 4: Table S2. Details of the detected viruses among cases with LRTI in which two viruses were detected in Biliran, Philippines, from March 2014 to June 2016. [file 12879_2022_7289_MOESM4_ESM.docx]

| Age in months | LRTI classification | Virus 1 | Virus 2 |
| --- | --- | --- | --- |
| 2 | Non-severe | RV-A | PIV-3 |
| 2 | Severe | RV-A | RSV-untyped |
| 3 | Non-severe | RV-A | PIV-3 |
| 3 | Non-severe | RV-A | PIV-1 |
| 3 | Non-severe | RV-B | RSV-B |
| 3 | Severe | RV-A | RSV-A |
| 4 | Non-severe | RV-C | AdV |
| 5 | Non-severe | RV-A | RSV-A |
| 5 | Non-severe | RV-C | RSV-B |
| 6 | Non-severe | RSV-B | PIV-3 |
| 6 | Severe | RV-B | RSV-B |
| 9 | Non-severe | MPV | IFV-B |
| 10 | Non-severe | RV-A | AdV |
| 12 | Undefined | RV-B | RSV-A |
| 14 | Non-severe | RSV-B | IFV-B |
| 14 | Severe | RV-B | RSV-B |

**Table S2.** Details of the detected viruses among cases with LRTI in which two viruses were detected in Biliran, Philippines, from March 2014 to June 2016.

Abbreviations: LRTI, lower respiratory tract infection; AdV, Adenovirus; RV, Rhinovirus; RSV, Respiratory syncytial virus; MPV, Human metapneumovirus; PIV, Parainfluenza virus; IFV, Influenza virus.
